# The Immediate Effects of Intermittent Theta Burst Stimulation of the Cerebellar Vermis on Cerebral Cortical Excitability During a Balance Task in Healthy Individuals: A Pilot Study

**DOI:** 10.3389/fnhum.2021.748241

**Published:** 2021-11-12

**Authors:** Hui-Xin Tan, Qing-Chuan Wei, Yi Chen, Yun-Juan Xie, Qi-Fan Guo, Lin He, Qiang Gao

**Affiliations:** ^1^West China Hospital, Sichuan University, Chengdu, China; ^2^Department of Rehabilitation Medicine, West China Hospital, Sichuan University, Chengdu, China

**Keywords:** intermittent theta-burst stimulation, cerebellar vermis, balance, functional near-infrared spectroscopy, transcrancial magnetic stimulation (TMS)

## Abstract

**Objective:** This pilot study aimed to investigate the immediate effects of single-session intermittent theta-burst stimulation (iTBS) on the cerebellar vermis during a balance task, which could unveil the changes of cerebral cortical excitability in healthy individuals.

**Subjects:** A total of seven right-handed healthy subjects (26.86 ± 5.30 years) were included in this study.

**Interventions:** Each subject received single-session iTBS on cerebellar vermis in a sitting position.

**Main Measures:** Before and after the intervention, all subjects were asked to repeat the balance task of standing on the left leg three times. Each task consisted of 15 s of standing and 20 s of resting. Real-time changes in cerebral cortex oxygen concentrations were monitored with functional near-infrared spectroscopy (fNIRS). During the task, changes in blood oxygen concentration were recorded and converted into the mean HbO_2_ for statistical analysis.

**Results:** After stimulation, the mean HbO_2_ in the left SMA (*P* = 0.029) and right SMA (*P* = 0.043) significantly increased compared with baseline. However, no significant changes of mean HbO_2_ were found in the bilateral dorsolateral prefrontal lobe (*P* > 0.05).

**Conclusion:** Single-session iTBS on the cerebellar vermis in healthy adults can increase the excitability of the cerebral cortex in the bilateral supplementary motor areas during balance tasks.

**Clinical Trial Registration:** [www.ClinicalTrials.gov], identifier [ChiCTR2100048915].

## Introduction

Balance is one of the most a critical function that supports the normal activities of daily life in humans. Maintaining balance requires a complex integration and coordination of multiple systems (such as the vestibular system, visual system and auditory system) in the body (Ataullah and Naqvi, 2021). The cerebellum is strongly involved in the integration process associated with balance in the central nervous system (CNS) (Nashef et al., 2019), as demonstrated by previous functional magnetic resonance imaging studies and clinical trials (Maurer et al., 2016; Esterman et al., 2017). According to the functional divisions of the cerebellum, the three functional areas include the cerebrocerebellum, the spinocerebellum and the vestibulocerebellum. These three functional areas all play a vital role in the process of maintaining balance and motor control. In the spinocerebellum, the cerebellar vermis plays a key role in balance and motor control. Recent evidence suggests that the cerebellar vermis is critical for maintaining equilibrium and coordinating speech, eye and body movement because the vermis provides information regarding sensations along the extremities, as well as the different stimuli that pertain to balance, visual and auditory processes (Fujita et al., 2020). In addition, the cerebellar vermis participates in anticipatory postural adjustment and compensatory postural adjustment to maintain balance during functional activities (Richard et al., 2017). Study has displayed patients with lesions in the vermis mainly exhibit balance dysfunction (Harris et al., 2018), while patients with lesions in the cerebellar hemispheres mainly exhibit global coordination dysfunction (Carass et al., 2018; Maas et al., 2020).

Except for the important role of cerebellum in balance, cerebral cortex is also involved. Previous studies illustrated that the supplementary motor area (SMA) and the dorsal lateral prefrontal cortex (DLPFC) are crucial during balance tasks (Richard et al., 2017; Harris et al., 2018; Fernandez et al., 2020). Richard et al. (2017) pointed out that the SMA plays an important role in the initiation of gait and the initiation of standing on one leg, which is related to maintaining stability during change of posture. Other studies have also confirmed the results obtained by Richard et al. (Mihara et al., 2012; Dale et al., 2019). Moreover, the increase in HbO_2_ related to postural disturbance in the contralesional SMA is significantly correlated with the increase in balance function measured by the Berg balance scale, and the postural disturbance-related changes in HbO_2_ signals in the bilateral SMA in stroke patients are positively correlated (Fujimoto et al., 2014). Studies also demonstrated that increased HbO_2_ was positively correlated with cortical excitability and functional improvement (Liu et al., 2018; Kinoshita et al., 2019). In addition, studies have demonstrated that intercortical connections exist between the DLPFC and primary motor cortex (M1). The connections from the DLPFC to the M1 transfer crucial information for the execution of motor output (Cao et al., 2018). The results from Teo et al. (2018) highlighted the involvement of the DLPFC in maintaining postural control. Study also demonstrated that the prefrontal cortex (PFC) and temporal-parietal regions were engaged during active balancing process, which was thought to be involved with allocation of attentional demands in standing postural control (Mihara et al., 2008). The non-invasive brain stimulation (NIBS) was applied to the DLPFC in patients with Parkinson’s disease and balance dysfunction, and balance function improved after intervention (Lattari et al., 2017). The results of a fNIRS study demonstrate that after external disturbances in healthy subjects, the bilateral DLPFC undergoes significant activation, which also indicates that the DLPFC also participates in maintaining balance (Mihara et al., 2012).

In current understandings, the process of maintaining balance involves the close cooperation between cerebellum and the cerebral. Some studies have established a strong anatomical and functional connections between cerebellum and M1 cortex through cerebellar-thalamus-M1 circuit (Coffman et al., 2011; Maurer et al., 2016). It is well worthy noted that previous study showed that the cerebellar vermis is a target of extensive projections from motor areas of cerebral cortex, which are involved in the regulation of whole-body posture and locomotion (Coffman et al., 2011). Additionally, anatomical experiment on monkey brains have shown that some cortical regions, especially the prefrontal cortex, receive projections from the cerebellum (Dale et al., 2019). Furthermore, Cho et al. (2012) pointed that the cerebellum connects not only to the M1 area and SMA but also to the DLPFC.

In recent years, NIBS of the cerebellum has also been a research hotspot (Benussi et al., 2020). There are a number of NIBS studies on the cerebellar hemisphere; however, studies of cerebellar vermis are relatively rare. Intermittent theta burst stimulation (iTBS), a type of repetitive transcranial magnetic stimulation (rTMS), which serves as a NIBS method, has been proved to have the positive effects on neuroplasticity and central nervous system excitability (Cotoi et al., 2019; Katagiri et al., 2020). It exhibits long lasting effects compared with traditional TMS with shorter stimulation period than traditional TMS (Kim et al., 2015; Hurtado-Puerto et al., 2020). Studies also demonstrated that iTBS can also modulate corticospinal excitability (Li et al., 2020). It is well known that regional hemodynamic responses are associated with cortical brain activation (Kinoshita et al., 2019). Recently, the use of fNIRS has become more widespread because the fNIRS system provides cortical brain activation information by measuring hemodynamic changes non-invasively (Kinoshita et al., 2019). Moreover, the cortical neural activity results obtained from fNIRS were similar to that from functional MRI (fMRI). The fNIRS is a useful tool that has been applied to record brain activation during balance tasks in previous study (Hoppes et al., 2020). Previous works by our research group have demonstrated that iTBS of the cerebellar hemisphere could improve balance and gait in patients with cerebral stroke. However, no significant changes in cortical excitability were observed (Liao et al., 2021).

Based on the extensive studies examining the relationship between cerebellum and cortex during balance tasks with the help of fNIRS, herein this study aimed to use fNIRS to explore the changes in cortical activation in the cerebral cortex (SMA and DLPFC) after iTBS of the cerebellar vermis during balance tasks. We hypothesized that activation of the SMA and DLPFC regions may increase after iTBS stimulation compared to baseline.

## Materials and Methods

### Subjects

A total of seven healthy volunteers were included in this study. The inclusion criteria were as follows: (1) age 18 to 35 years old (Aloraini et al., 2019), (2) free from neurological and psychiatric disorders, (3) right-handed dominance, (4) body mass index from 18.5 to 23.9 (Corp et al., 2018), and (5) signed informed consents. The exclusion criteria were as follows: (1) having suffered from neuropsychological diseases (depression, anxiety, schizophrenia and so on) in the past, (2) having a history of drug abuse (drugs that are potential hazards for rTMS, especially drugs that can lower seizure threshold) (Rossi et al., 2009), (3) individuals with musculoskeletal disorders, especially disorders in the lower limb and trunk, (4) subjects with vestibular deficits and (5) participated in other TMS experiments without reporting TMS exclusion criteria.

### Intermittent Theta Burst Stimulation

**FIGURE 1:**
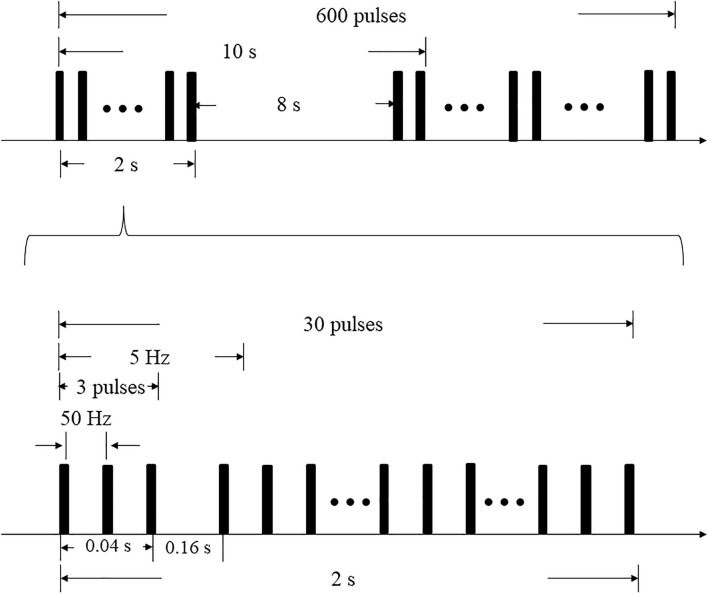
iTBS protocol.

A CCY-I rapid magnetic stimulator, which was connected to a double-cone coil (YIRUIDE Medical, China) was used to stimulate the cerebellar vermis for single-session iTBS. The coil type was a V1320T double-cone coil. The parameters of the coil were listed as follow: (1) outside diameter: Φ130 mm; (2) inside diameter: Φ70 mm; (3) distance between the two highest points at the top of the coil: 200 mm; (4) angle formed by the two conical coils: 120°. The targeted point was located 1 cm below the “inion” (the highest point of the external occipital protuberance) i.e., midline cerebellum (Cattaneo et al., 2014; Garg et al., 2016), and the coil was positioned tangentially to the scalp. When applying iTBS, the stimulator output intensity was set to the level of individual’s maximum tolerated intensity (MTI) (Spampinato et al., 2020). The iTBS protocol began with a two-second burst train (totally 30 pulses), which repeats every 10 s. Each burst train consisted of ten triplet pulses with an inter-burst interval of 0.16s, thus the triplets fire at a rate of 5 Hz (Figure 1). Overall, each subject received 600 stimuli during a single-session iTBS (Huang et al., 2005). The total duration of the iTBS protocol was 3′20″ (Cao et al., 2018).

### fNIRS Measurement

**FIGURE 2:**
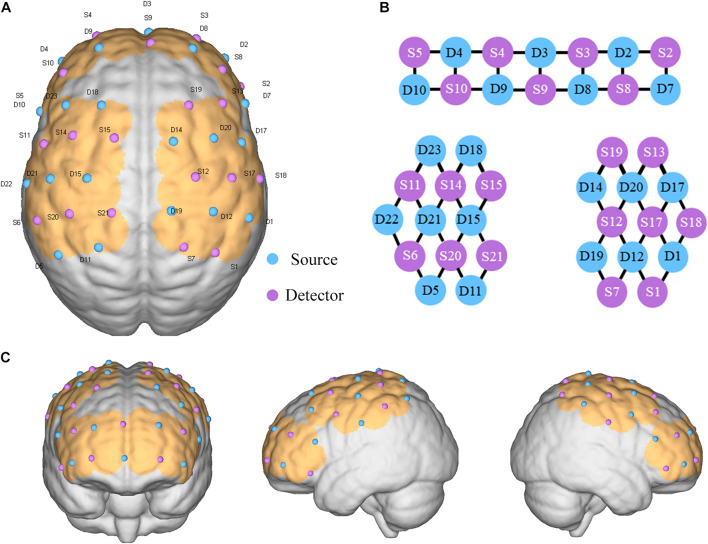
Placement of optode. **(A)** Optode set arrangement with numbers displayed on a three-dimensional model of the brain (ICBM 152 Non-linear Atlases, version 2009). **(B)** Two-dimensional distribution of the light source and detection point. **(C)** Cerebral cortex where channels are covered.

A multichannel fNIRS system (NirScan, HuiChuang, China) was used to record changes of HbO_2_ in the cortex of the SMA and DLPFC. The wavelengths were set to 730 and 850 nm. Data were sampled at a frequency of 10 Hz (Lu et al., 2019). Fifty five channels (defined as the midpoint of the corresponding light source-detector pair) were established, with 20 light sources and 20 detectors for measurement. These channels were symmetrically distributed in the left and right cerebral hemispheres of the participants. The center of the middle probe set row was placed at approximately FPz, according to the 10/20 international system (Hu et al., 2019). The optodes were positioned over the left and right DLPFC (L-DLPFC: S10-D4, S10-D9, S11-D23 and S14-D23; R-DLPFC: S8-D2, S8-D8, S13-D17 and S13-D20) and the left and right SMA (L-SMA: S14-D15 and S15-D15; R-SMA: S12-D14 and S12-D20) (Figure 2).

### Experimental Procedure

**FIGURE 3:**
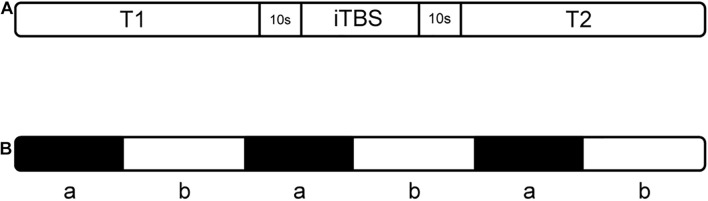
The experimental procedure **(A)** includes three phases; “T1” and “T2” indicate balance tasks, **(B)** balance task in T1 and T2: “a” means standing on the right leg for 15 s, “b” means resting for 20 s while standing.

There were three phases of this experimental procedure (Figure 3A). During the trial, the humidity and temperature of the environment were kept stable, and the personnel present remained quiet. The three phases comprised a session of iTBS (while sitting) and balance tasks (while standing), which were identical before and after the stimulus (T1 and T2 phases). For the balance tasks, we set the process and the prompt sound at the corresponding time point in NirScan. During the balance task, participants were asked to stand with their left leg raised three times separate (Figure 3B). To minimize the error of the fNIRS recording process, which is usually due to postural changes, we allowed 10 s for sit-stand postural changes before and after stimulation (Liu et al., 2018; Koch et al., 2019). Throughout the three phases, real-time HbO_2_ data was collected using fNIRS. In addition, we ensured that the temperature and humidity in the test process were basically unchanged, and everyone in the room (subject and assessor) remained silent throughout the entire test period (Bu et al., 2019).

### Data Processing

The NirSpark (HuiChuang, China) software package was used to analyze the fNIRS data. First, light intensity was converted to optical density (OD). Then, motion artifacts were corrected the moving standard deviation and cubic spline interpolation method. A bandpass filter with cutoff frequencies of 0.01–0.1 Hz was then applied to remove physiological noise (respiration, cardiac activity and low-frequency signal drift). Finally, the filtered OD signal was converted to Delta-HbO2 and Delta-HHB according to the modified Beer-Lambert law. After that, we averaged the HbO_2_ data of the three periods standing on one leg during T1 and T2. The mean value in the range of 10–15 s (relative to condition onset) was used for further statistical analysis.

### Statistical Analysis

The mean HbO_2_ values of each channel for 7 subjects before and after stimulation were imported into SPSS version 24.0 (IBM Corp) for analysis. The normality of the data was assessed using the Kolmogorov–Smirnov test. Except for channels S12-D14, the data from all channels passed the Kolmogorov–Smirnov test, which presented as the means (± standard deviations, SDs). The rank sum test was used for data from channels S12-D14, the data are presented as the medians (interquartile ranges, IQRs). For the DLPFC, the data from all channels passed the Kolmogorov–Smirnov test. A paired *t*-test was used for the data of the remaining channels, and presented as the means (± standard deviations, SDs). A difference with *P* < 0.05 was considered statistically significant.

### Ethics Committee

Ethical approval was obtained from the biomedical ethics committee of West China Hospital at Sichuan University. The protocol of this study was registered with the Chinese Clinical Trial Registry (registration number: ChiCTR2100048915).

## Results

**FIGURE 4:**
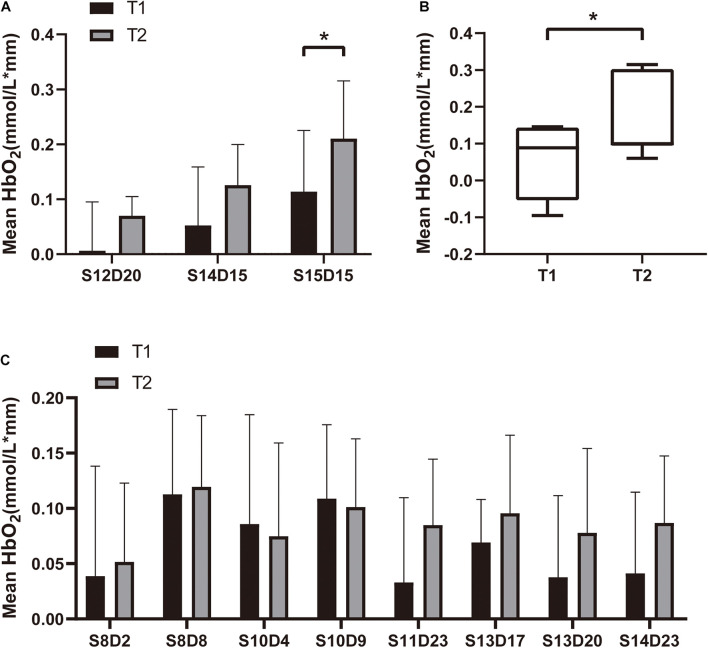
Mean HbO_2_ for each channel in the **(A)** SMA (S12D20, S14D15 and S15D15), **(B)** SMA (S12D14), and **(C)** DLPFC. We plot the mean with SD in panels **(A,C)**, and median with min to max in panel **(B)**. *P < 0.05.

**FIGURE 5:**
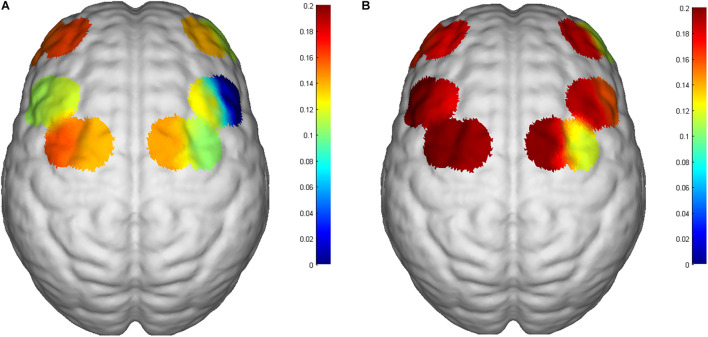
Changes in mean HbO2 of the 6th subject. **(A)** Before iTBS and **(B)** post iTBS. Significant activation in the SMA in the post-iTBS phase relative to baseline. The number on the left of the color bar stands for mean HbO2, which means that the larger the mean HbO2 value of a channel, the closer the color is to red; otherwise, the closer it is to blue.

All subjects tolerated the trial, with no adverse events (Table 1). Mean HbO_2_ significantly increased in channels S12-D14 (*P* = 0.043, *Z* = 2.028) and S15-D15 (*P* = 0.029, *t* = 2.849) at T2 compared with T1 (Table 2 and Figure 4). There was no difference in any channel in the DLPFC (*P* > 0.05) (Table 2 and Figure 4). In addition, we also found that the mean changes in HbO_2_ in the bilateral brain region before and after stimulation were obvious through the distribution map of mean HbO_2_ (Figure 5).

**TABLE 1:** Characteristics of the subjects (*N* = 7).

Subjects	Gender	Hand dominance	Age (year)	Height (cm)	Weight (kg)
1	M	Right	20	170	67
2	M	Right	34	169	60
3	M	Right	26	182	65
4	M	Right	27	165	78
5	M	Right	24	187	85
6	M	Right	22	172	64
7	M	Right	35	160	55
All	M	Right	26.86 ± 5.30^a^	172.14 ± 8.71^a^	67.71 ± 9.62^a^

*^*a*^Mean ± standard deviation.*

**TABLE 2:** Mean HbO_2_ for each channel at T1 and T2.

Brain area	Channel	T1	T2
**SMA**			
R-SMA	S12-D14	0.09 (0.2)	0.1 (0.21)*
	S12-D20	0.010.09	0.070.03
L-SMA	S14-D15	0.050.11	0.130.07
	S15-D15	0.110.11	0.210.10*
**DLPFC**			
R-DLPFC	S8-D2	0.0390.1	0.050.07
	S8-D8	0.110.07	0.120.64
	S13-D17	0.070.04	0.100.07
	S13-D20	0.040.07	0.080.08
L-DLPFC	S10-D4	0.090.1	0.070.08
	S10-D9	0.110.07	0.100.06
	S11-D23	0.030.08	0.850.06
	S14-D23	0.040.07	0.090.06

*P < 0.05.

## Discussion

In this study, we found that single-session iTBS of the cerebellar vermis could increase the concentration of HbO_2_ in the SMA region during the balance task, but no significant increase was found in the DLPFC.

After a single-session of iTBS on cerebellar vermis, the increments of HbO2 in the SMA region indicated an excitability increase in the SMA cortex. This result indicates a potential connection between the cerebellar vermis and the SMA, which is consistent with previous studies (Coffman et al., 2011; Franca et al., 2018). Animal study has demonstrated that cortical motor areas are a source of input to the cerebellar vermis, especially lobules VB–VIIIB (Coffman et al., 2011). One of these areas is the SMA. In our study, it was interesting that the excitability of the bilateral SMA increased during the balance task after iTBS on cerebellar vermis, which may indicate that the cerebellar vermis and bilateral SMA are involved in neuromotor control and balance processes. A study conducted by Fujimoto et al. (2014) showed that increased cortical excitability in the bilateral SMA regions was positively correlated with improved balance function (Fujimoto et al., 2014), some other studies obtained the same results (Kinoshita et al., 2019). Therefore, the cerebellar vermis may be a new target for iTBS stimulation to influence balance function and postural control in humans, but more formal studies are needed to prove this hypothesis.

In this study, we found no significant changes in HbO_2_ in the DLPFC. However, previous studies have demonstrated that the DLPFC is also involved in postural control (Taube et al., 2015), coordinating with the cerebellar vermis (Farzan et al., 2016). Furthermore, the DLPFC participates in planning actions by providing flexibility among already acquired solutions (Torriero et al., 2007). Early results indicated that, in addition to the long-established role of the cerebellum in motor coordination, the cerebellum appears to be involved in the central integration of cognition and emotion (Demirtas-Tatlidede et al., 2011; Cho et al., 2012). The connection between the cerebellum and the contralateral motor cortex is close and important. In addition to the M1 area, the cerebellum also projects to the DLPFC (Maas et al., 2020). Our study result is inconsistent with the results of previous studies (Mihara et al., 2012). A possible explanation for this result might derived from the small sample size. The balance task is very simple, which does not involve much cognitive function or motor integration, may be another possible explanation (Torriero et al., 2007). Moreover, the DLPFC, along with its association connections, constitutes a potential cortical network for visual reaching (Johnson et al., 1996). However, in our study, subjects were required to open their eyes while standing on a single leg to maintain balance, which may account for the lack of significant differences in the data.

We already know that these inter-connections between cerebellum and cerebral tissue are mainly bundles of white matter fibers with the following connective pathways: the cortico-ponto-cerebellar pathway and cerebello-thalamo-cortical (CTC) pathway (Iwata and Ugawa, 2005; Opie and Semmler, 2020). These neural pathways are functionally involved in motor coordination and cognitive functions (Bonnì et al., 2014). Moreover, previous studies have demonstrated that fibers from the cerebellar hemispheres could project to the contralateral cerebral cortex (Tramontano et al., 2020; Solanki et al., 2021). In addition, in right-handed individuals, the connection between the right cerebellum and the left M1 is usually stronger than that of the contralateral network (Schlerf et al., 2015). This implicates that we can explore the differences in cortical excitatory activation on both sides in future studies.

In the studies we reviewed, NIBS of the cerebellar vermis could be traced back to 1995. Hashimoto et al. used TMS on cerebellar vermis of humans to explore the relationship between the cerebellar vermis and eye saccades (Hashimoto and Ohtsuka, 1995). The results of their study demonstrated that the posterior medial cerebellum in humans is involved in controlling the accuracy of visually guided saccades. Since then, due to neurophysiology of the connections between the cerebellum and different cortical layers of the cerebral cortex drew much attention, studies of NIBS of the cerebellum have gradually increased in different fields. For balance function and postural control in males, Cha YH indicated that cTBS on occipital cortex or the cerebellar vermis could effectively reduce the wobbling vertigo of Mal de Debarquement syndrome and may yield long-term benefits for patients (Cha et al., 2019). In 2020, a feasibility study found an association between cerebellar lobular mean electric field strength and changes in quantitative gait parameters after a single cerebellar transcranial direct-current stimulation (tDCS) session in chronic stroke patients (Kumari et al., 2020). In addition, the cerebellar vermis is involved in directing the postural response to a vestibular perturbation, which supports our hypothesis that the cerebellar vermis could be a target of NIBS to affect balance function and walking. Additionally, a study demonstrated that cTBS stimulating cerebellar vermis could lead to a significant influence on balance function as body sway increases (Colnaghi et al., 2017). For other effects on humans, Sasegbon et al. (2021) found that TMS on cerebellar vermis has an inhibitory effect on pharyngeal cortical activity and swallowing. Moreover, Argyropoulos et al. (2011) demonstrated that TBS of the right neocerebellar vermis can selectively disrupt the practice-induced acceleration of lexical decisions. As pointed out in neurophysiological research, the cerebellar vermis may be involved in the regulation of a series of non-physical functions (Van Overwalle et al., 2020). iTBS of the cerebellar vermis could result in the negative symptoms of schizophrenia patients. Similarly, non-invasive brain stimulation of the cerebellar vermis can also relieve symptoms of depression (Escelsior et al., 2019).

Regarding the coils we used, previous studies presented mostly used a figure-8 coil for iTBS (Franca et al., 2018). However, the latest research recommends a double-cone coil (Spampinato et al., 2020) because the use of a double-cone coil can achieve deeper stimulation effects in the cerebellar vermis. The safety of the double-cone coil in iTBS is guaranteed (Spampinato et al., 2020). In summary, stimulation of the cerebellar vermis with iTBS can increase the cortical excitability of the bilateral SMA, but the changes in the DLPFC are not significant and still need further exploration in formal studies.

## Limitations

There are some limitations of our pilot study. Firstly, the major limitation of our study is that we did not used behavioral assessments to prove whether iTBS could improve balance function. However, more behavioral assessments will be performed in our future formal studies to determine whether iTBS of the cerebellar vermis could improve balance function. Second, the study had a small sample size and was limited to healthy volunteers, and a larger sample size is needed in future studies. The focus of future trials should be the inclusion of more patients. Third, our trial did not use navigational equipment, which may have reduced the accuracy of iTBS processes. In future studies, we will include navigational devices in experiments to improve the accuracy of iTBS on cerebellar vermis. Besides, the statistical tests in our study were not Bonferroni-corrected, which may increase the probability of type I error. Furthermore, we used only a single-session of iTBS of the cerebellar vermis in this study, and the long-term effects of iTBS of the cerebellar vermis will be explored in formal experiments in the future.

## Conclusion

In this study, we found that a single-session iTBS of the cerebellar vermis in healthy adults can increase the excitability of the cerebral cortex in the bilateral SMA during the balance tasks. Therefore, the iTBS on the cerebellar vermis may be a potentially effective intervention for improving balance function for the stroke patients with balance disorders.

## Data Availability Statement

The original contributions presented in the study are included in the article/supplementary material, further inquiries can be directed to the corresponding author/s.

## Ethics Statement

The studies involving human participants were reviewed and approved by Institutional Review Board of West China Hospital, Sichuan University. The patients/participants provided their written informed consent to participate in this study.

## Author Contributions

H-XT and Q-CW devised the project under the supervision of QG and drafted the manuscript. YC, Y-JX, Q-FG, and LH conducted the data collection and data analysis. All authors provided critical feedback and helped shape the research, analysis, and manuscript.

## Conflict of Interest

The authors declare that the research was conducted in the absence of any commercial or financial relationships that could be construed as a potential conflict of interest.

## Publisher’s Note

All claims expressed in this article are solely those of the authors and do not necessarily represent those of their affiliated organizations, or those of the publisher, the editors and the reviewers. Any product that may be evaluated in this article, or claim that may be made by its manufacturer, is not guaranteed or endorsed by the publisher.
